# Health-Related Quality of Life of People with Self-Reported Hypertension: A National Cross-Sectional Survey in China

**DOI:** 10.3390/ijerph16101721

**Published:** 2019-05-16

**Authors:** Qiang Yao, Chaojie Liu, Yaoguang Zhang, Ling Xu

**Affiliations:** 1 School of Political Science and Public Administration, Wuhan University, Wuhan 430072, China; yaoqiang@whu.edu.cn; 2 School of Psychology and Public Health, La Trobe University, Melbourne 3086, Australia; c.liu@latrobe.edu.au; 3 Institute of health Research, Wuhan University, Wuhan 430072, China; 4 Center for Health Statistics and Information, National Health Commission, Beijing 100810, China; 5 Health Human Resources Development Center, National Health Commission, Beijing 100044, China

**Keywords:** hypertension, health-related quality of life, EQ-5D-3L, National Health Services Survey, China

## Abstract

This study aimed to determine the health-related quality of life (HRQoL) of people with self-reported diagnosed hypertension and its determinants in China. Data was obtained from the 5th National Health Services Survey. The HRQoL of the respondents who were 15 years or older was assessed with the EQ-5D-3L utility index and visual analogue scale (VAS), and compared between those with (*n* = 30,063) and without (*n* = 158,657) self-reported hypertension. Multivariate logistic regression, Tobit regression, and linear regression models were established to identify predictors of HRQoL. A difference of half standard deviation was deemed as minimal clinically important difference (MCID) for the utility index (0.03). The respondents with self-reported hypertension were more likely to report problems in the five dimensions (Adjusted Odds Ratio = 1.43–1.70) of the EQ-5D-3L, resulting in a significant lower utility index (β = −0.04) and VAS scores (β = −3.22) compared with those without self-reported hypertension, and the difference of the utility index exceeded MCID. In the respondents with self-reported hypertension, higher utility index and VAS scores were found in those who were female, younger, married, employed, smoking, drinking, exercising regularly, absent from comorbidity, resided in the eastern developed region, had normal body mass index, higher levels of education, and income. Hypertension management programs were associated with higher utility index (β = 0.01) and VAS scores (β = 1.02). Overall, hypertension is associated with lower HRQoL. Higher socioeconomic status and participation in management programs for chronic conditions are independent predictors of higher HRQoL of hypertensive people. This study provides a national representative estimate on the HRQoL of hypertensive people in China, which can be used for calculating the burden of hypertension.

## 1. Introduction

Hypertension is the biggest single contributor to global burden of disease (GBD). Over the past few decades, the prevalence of hypertension increased substantially, resulting in significant loss of disability-adjusted life-years (DALYs) [1]. It was estimated that globally there were 1.13 billion adults with hypertension in 2015, compared with just 594 million in 1975 [2]. The prevalence of hypertension increased from 17.31% in 1990 to 20.53% in 2015, leading to an increase of annual deaths from 97.9 to 106.3 per 100,000 persons, and an increase of loss of DALYs from 95.9 million to 143.0 million worldwide [3,4]. Hypertension has become a leading cause of GBD, as an important risk factor of cardiovascular disease, stroke, and chronic kidney disease [1,5,6].

China, India, Russia, Indonesia, and the United States account for more than half of the global DALYs related to hypertension [3,4]. Hypertension is the leading cause of death and disability in China [5]. Several large population surveys in China revealed that the prevalence of hypertension increased significantly over the past few decades, rising from 18.0% in 2002 to 27.8% in 2013 for people aged 18 years and above [7,8]. Hypertension caused more than 2 million deaths in China in 2010, contributing to 24.6% of all deaths in the country [9].

Hypertension has even more profound impacts on the physical, psychological, social, and emotional functioning of the patients [10]. It was estimated that hypertension led to 10,667 loss of DALYs per 100,000 population in China: 78.3% as a result of functioning impairments and 21.7% from premature deaths [11]. Hypertension is often left untreated [12]. In China, fewer than one third of hypertensive people were aware of their condition and less than 10% of hypertensive people had their blood pressure properly controlled [8,11,13].

It is important to assess health-related quality of life (HRQoL) of hypertensive people. HRQoL can serve as a foundation for calculating DALYs, taking into consideration the impact of hypertension on the physical, psychological, social, and spiritual wellbeing of the patients from the perspective of the patients themselves [14]. Such measurements are often used for guiding policy development [15]. International evidence consistently shows that hypertension lowers HRQoL [12,14,16,17,18,19,20,21,22,23]. However, the size of its effect varies considerably across countries due to differences in sociodemographic and cultural characteristics of the patients [24].

Previous studies on the HRQoL of hypertensive people in mainland China are limited. Wang et al. [25] and Xu et al. [26] assessed the HRQoL of hypertensive people in Shanghai (aged 35–75 years) and Chongqing (aged 45–53 years), respectively, using the 36-item Short Form (SF-36). Hypertension was found to be associated with poorer physical functioning, but it was found to be less significant in the mental health component. Zhang et al. [27] and Pan et al. [28] used the EQ-5D-3L instrument to estimate the utility index of hypertensive people in Shandong (aged 18 years and above) and Suzhou (aged 60 years and above), respectively. The results were inconsistent. Pan et al. [28] didn’t find a utility index difference, contrasting the findings by Zhang et al. [27]. These studies suffered from some common limitations. Firstly, the samples were small, targeting local populations and selected age groups only. Secondly, only a few studies converted the HRQoL results into a utility index, which is essential for estimating DALYs and health economics analyses [10,27,28]. Thirdly, the national population-preference based value sets for the EQ-5D-3L was only made available in 2018 [29]. The previous studies either borrowed the value sets from other countries or used the value sets developed by Liu [30] based on a small sample from four big cities.

This study aimed to determine the HRQoL of people with self-reported diagnosed hypertension in a national representative sample in China. To the best of our knowledge, this study is the first of its kind [31], estimating the EQ-5D-3L utility index for hypertensive people based on a value set derived from a large national representative sample [29]. In this study, we also identified demographic, socioeconomic, behavioral, and health services factors associated with the HRQoL of people with hypertension.

## 2. Materials and Methods

### 2.1. Study Design and Data Source

Data was extracted from the 5th National Health Services Survey (NHSS). The NHSS is a cross sectional household questionnaire survey conducted in a national representative sample in China every five years. The surveys were overseen by the Centre for Health Statistics Information under the national health authority. The 5th NHSS was conducted in September 2013 [32]. A standard protocol and strict quality control procedures applied. Data were collected by trained local health workers through face-to-face interviews. Each field site had a survey supervisor who revisited 5% of the participating households. Overall, 97.7% of the repeated surveys were consistent with the original ones in the examined key questions in the 5th NHSS. The Myer’s index (2.55), DELTA dissimilarity coefficient (0.085), and the GINI concentration ratio (0.0525) indicated a national representativeness of the sample [32].

### 2.2. Setting and Sample

A four-stage stratified cluster random sampling method was adopted to select participants. A total of 93,600 households were sampled from 1560 communities/villages in 780 sub-districts/townships from 156 counties/districts across all 31 provinces in mainland China. All of the members in the participating households were interviewed individually. A total of 273,688 questionnaires were completed.

Data collected in the questionnaire survey covered the demographic and socioeconomic characteristics of the respondents, their health behaviors, health status, and use of health services [33,34,35]. These included the EQ-5D-3L, which was applied to those who were 15 years and older [36,37,38]. In this study, the returned questionnaires containing missing data in age, gender, and the EQ-5D-3L were excluded. This resulted in a sample of 188,720 for data analyses.

Hypertensive people were identified through the questions in relation to chronic conditions. The respondents were asked whether they have been diagnosed with hypertension by a doctor, which only captured those who were aware of their conditions and sought medical diagnoses [8,11,13]. Of the study sample, 30,063 reported hypertension, compared with 158,657 reporting no diagnosed hypertension.

### 2.3. Dependent Variables

The HRQoL of the respondents was assessed using the EQ-5D-3L, a generic instrument developed by the EuroQol Group in 1990. Previous studies have confirmed its reliability and validity in mainland China [39,40]. Three indicators were generated to reflect the HRQoL of the respondents: (1) percentage of respondents reporting problems in the five dimensions; mobility (MO), self-care (SC), usual activity (UA), pain/discomfort (PD), and anxiety/depression (AD). The three levels of measurements were recoded into two levels; with (moderate or extreme problem) and without (no problem) problems. (2) utility index; the combination of problems on the five dimensions related to each individual was converted into a utility index score (ranging from 0.170 to 1.000) based on the population preference-based value sets derived from the Time-Trade-Off (TTO) technique by Zhuo in 2018 [29]. (3) EQ-VAS score; the respondents rated their overall health on a visual analogue scale (VAS) ranging from 0 to 100, with a higher score indicating better perception of health. The EQ-5D-3L instruments used in the NHSS was an official self-complete paper version registered in the EuroQol group. However, its vertical VAS was rotated to a horizontal one to fit into the paper questionnaire for the NHSS [24,41].

### 2.4. Independent Variables

The selection of independent variables associated with the HRQoL of hypertensive people was guided by the World Health Organization (WHO) determinants of health model [42,43]. These variables were grouped into five clusters in line with the Dahlgren-Whitehead rainbow model [42], including biology and genetics, health behaviors, socio-economic characteristics, communities, and regions, and health policy and services.

Biology and genetics: Data collected in the NHSS included gender (male or female), age (15–24, 25–34, 35–44, 45–54, 55–64, 65–74, 75+), and body mass index (BMI). The BMI (kg/m^2^) was calculated as “weight (in kilograms) divided by the square of height (in meters)”. All of the data including body height and weight were self-reported from the survey participants. According to the WHO International BMI classification criteria [44,45], respondents were categorized into four groups: Underweight (BMI < 18.5), normal weight (18.5 ≤ BMI < 25.0), overweight (25.0 ≤ BMI < 30.0), and obese (BMI ≥ 30.0). The respondents were also asked whether they “have ever been diagnosed with any other chronic conditions by a doctor?” The co-existence of chronic conditions other than hypertension was labelled as comorbidity, such as diabetes, and rheumatoid arthritis etc.

Health behaviors: In the NHSS, respondents were asked to answer the following three questions: (1) “Do you smoke any tobacco products currently?” (yes or no); (2) “Over the last 12 months, have you ever drunk alcohol?” (yes or no); (3) “Over the last six months, how often do you exercise every week?”. A person who engaged in physical exercises at least once a week was deemed physically active [32].

Socio-economic characteristics: The socioeconomic status of respondents was measured by educational attainment (illiterate, primary school, junior middle school, senior middle school, university/college), employment (employed, retired, student, unemployed), marital status (single, married, divorced, widowed), and income ranking (<percentile 20, percentile 20–39, percentile 40–59, percentile 60–79, and ≥percentile 80 in terms of average household income per capita).

Communities and regions: Area of residency (urban vs rural) and geographic location (eastern developed, western undeveloped, central in between) were used to measure regional disparities [46,47].

Health policy and services: Patients with chronic conditions were encouraged by the Chinese government to register with a primary care team in the local community for systematic management of their conditions. This included regular monitoring of illness conditions (such as blood pressure), coaching on lifestyles, and advices on the use of medicines [48,49]. In the NHSS, hypertensive people were asked whether they received such management services: “Over the past three months, have any medical staff guided you on preventing and controlling hypertension?”. Regular health examinations are considered an important step for identifying and mitigating the risks of complications of chronic conditions [10]. In the NHSS, respondents were asked whether they received any health examination over the past 12 months prior to the survey.

### 2.5. Statistical Analysis

The percentage of respondents reporting problems on the five dimensions of the EQ-5D-3L and mean utility index and VAS scores were presented. Pearson χ² tests were employed to examine group differences in the percentage of reported problems. Student t tests and analysis of variance (ANOVA) were performed to examine group differences in utility index and VAS scores.

Multivariate regression models were established to determine the association between hypertension and HRQoL after adjustments for variations in other independent variables. We then performed multivariate regression analyses with the sample comprising hypertensive people only to explore factors associated with the HRQoL of hypertensive people. The regression analyses applied binary logistic regression models for the percentage of reported problems on the five dimensions, Tobit regression models for the utility index (bounded data), and linear regression models for the VAS scores. The robust method was used to estimate variance-covariance matrix (VCE) corresponding to the parameter estimates [10]. The statistical significance level was set at 0.05. All analyses were performed using STATA version 14.0 (SE) (StataCorp., College Station, TX, USA) for Windows.

In addition, group differences in the utility index were further assessed using the minimal clinically important difference (MCID) indicator. Previous studies estimated a MCID ranging from 0.033 to 0.074 for the EQ-5D utility index [18,50,51]. A difference of half standard deviation (SD) was usually deemed as a threshold of MCID [52], which was 0.03 in this study.

All procedures performed in studies involving human participants were in accordance with the ethical standards of the institutional and/or national research committee and with the 1964 Helsinki declaration and its later amendments or comparable ethical standards. The NHSS received ethics approval from the institutional review board of the Chinese National Bureau of Statistics (license number 2013-65). Additional informed consent was obtained from all individual participants.

## 3. Results

### 3.1. Characteristics of Respondents

In this study, 15.93% respondents reported diagnosed hypertension, which was comparable to findings of previous studies (ranging from 8.87% to 19.98%) [8,13,53,54]. Women and those who were older, widowed, retried or unemployed, had overweight/obesity, received less education, had lower income, resided in an urban area, came from the eastern developed region, and had comorbidities were more likely to report hypertension than others (*p* < 0.01). Hypertensive people were less likely to smoke, drink, and take regular exercise (Table 1).

### 3.2. Hypertension and HRQoL

Pain/discomfort was the most frequently reported problem: 25.96% in hypertensive people compared with 10.08% in those without self-reported diagnosed hypertension (*p* < 0.001). Problems in self-care were the least frequently reported: 8.01% in hypertensive people compared with 2.12% in those without self-reported diagnosed hypertension (*p* < 0.001) (Table 2).

**Figure 1:**
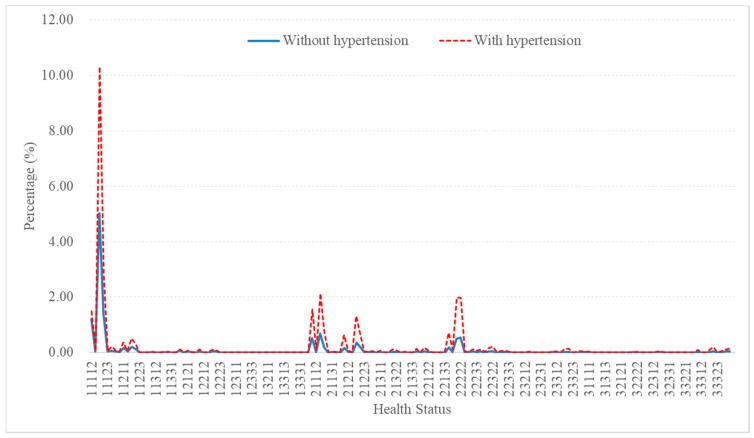
Distribution of health states (without full state 1.000) in people with and without self-reported diagnosed hypertension.

A total of 162 health states (a combination of problems on the five dimensions of the EQ-5D-3L) were reported and the majority reported no problem at all (“11111”): 68.06% in hypertensive people compared with 87.24% in those who did not report diagnosed hypertension (*p* < 0.001). In both groups, the most frequently reported state was moderate pain/discomfort (“11121”), followed by moderate problems in mobility and pain/discomfort (“21121”) and moderate problems on all five dimensions (“22222”). Overall, hypertensive people were more likely to report problems than those without self-reported diagnosed hypertension (Figure 1), with adjusted odds ratio (AOR) ranging from 1.43 (95% CI 1.38–1.48) to 1.70 (95% CI 1.59–1.81) in the logistic regression models (Table 3).

The hypertensive people had a mean utility index score of 0.964 (SD = 0.088), significantly lower than that (0.989 ± 0.047) of those without self-reported diagnosed hypertension (*p* < 0.001, Table 1). The significance of the difference was confirmed in the multivariate regression model (β = −0.04, Table 4). The difference (0.72 of SD) also exceeded the threshold of MCID (0.03).

The hypertensive people had a mean VAS score of 73.04 (SD = 15.11), significantly lower than that (82.40 ± 12.94) of those without self-reported diagnosed hypertension (Table 1). The significance of the difference was confirmed (β = −3.22) in the multivariate regression model (Table 4).

### 3.3. Factors Associated with HRQoL of Hypertensive People

The female respondents with self-reported diagnosed hypertension were less likely to report problems in mobility (AOR = 0.66), self-care (AOR = 0.57), and usual activities (AOR = 0.60) compared with their male counterparts (Table 5), resulting in a higher utility index (β = 0.02) and VAS score (β = 0.59, Table 6). Those aged 75 years or older with self-reported diagnosed hypertension were more likely to report problems in usual activities (AOR = 7.92, Table 5) and had a lower utility index (β = −0.17) and VAS score (β = −15.89, Table 6) than their younger counterparts. Although the hypertensive (self-reported) respondents with a higher body weight were less likely to report problems and had higher utility index and VAS scores than the underweighted in general (Table 5), those with obesity had a similar utility index score as the underweighted (Table 6). The hypertensive (self-reported) respondents with comorbidity were more likely to report problems on all of the five dimensions (AOR = 2.18–2.90, Table 5) compared with those without comorbidity, resulting in a lower utility index (β = −0.10) and VAS score (β = −6.99, Table 6). However, the gender and BMI differences in the utility index did not reach the MCID threshold.

The hypertensive (self-reported) respondents with higher educational attainment were less likely to report problems on all of the five dimensions (AOR = 0.55–0.89, Table 5), resulting in a higher utility index (β = 0.02–0.05) and VAS score (β = 1.62–2.16, Table 6). Similarly, the hypertensive (self-reported) respondents with higher income levels were less likely to report problems on all of the five dimensions (AOR = 0.60–0.89, Table 5), resulting in a higher utility index (β = 0.02–0.04) and VAS score (β = 1.63–3.31, Table 6). The unemployed were more likely to report problems on all of the five dimensions (AOR = 1.62–3.25, Table 5) and had a lower utility index (β = –0.08) and VAS score (β = –4.41, Table 6) than the employed. The married were less likely to report problems on all of the five dimensions (AOR = 0.63–0.87, Table 5) and had a higher utility index (β = 0.03) and VAS score (β = 3.71, Table 6) than those singles.

Compared with the hypertensive (self-reported) respondents living in the eastern region, those living in the west were more likely to report problems on all of the five dimensions (AOR = 1.45–1.92, Table 5) and had a lower utility index (β = −0.04) and VAS score (β = −3.78, Table 6). Rural hypertensive (self-reported) respondents were more likely to report problems on all of the five dimensions (AOR = 1.10–1.22, Table 5) and had a lower utility index (β = −0.01, Table 6) compared with their urban counterparts. However, the urban-rural difference in the utility index (β = −0.01) did not reach the MCID threshold. In addition, the rural hypertensive (self-reported) respondents had a higher VAS score (β = 0.67, Table 6).

Smoking (AOR = 0.69–0.89) and drinking (AOR = 0.52–0.82) were associated with a lower likelihood of reporting problems on the five dimensions (Table 5). Higher utility index and VAS scores were found in the hypertensive (self-reported) respondents who smoked and drunk (Table 6). The hypertensive (self-reported) respondents who exercised regularly were less likely to report problems on all of the five dimensions (AOR = 0.36–0.71, Table 5) and had a higher utility index (β = 0.06) and VAS score (β = 2.73, Table 6) than those who did not.

The hypertensive (self-reported) respondents who enrolled in the management programs for chronic conditions (AOR = 0.87–0.98) and received health examinations over the past year (AOR = 0.70–0.96) were less likely to report problems on all of the five dimensions (Table 5) and had higher VAS scores (Table 6). However, the differences in the utility index failed to reach the MCID threshold.

## 4. Discussion

This study provides a HRQoL profile for people with self-reported diagnosed hypertension and its related factors in China using the EQ-5D-3L instrument based on a nationally representative sample. We found that people with diagnosed hypertension have lower HRQoL than those without diagnosed hypertension and such a difference has reached the threshold of MCID.

Although the average utility index of the self-reported diagnosed hypertensive people in China appears high (0.964) compared with those in many other countries (ranging from 0.470 to 0.910) [12,17,18,21,22,55,56], it is important to note that the EQ-5D-3L population norms in China are also higher than those in other countries [57]. This may be caused by the lower health expectation of the Chinese people and their higher tolerance to the influence of health problems, especially in those living in rural areas.

In China, people who reported diagnosed hypertension had a lower utility index than those without diagnosed hypertension. The effect size (−0.04) is comparable to that in other countries, such as the US (−0.038 < β < 0) [18], Finland (−0.021) [17], Singapore (−0.04) [56], and Korea (−0.075) [12]. Such a comparable effect size is also evident in VAS score differences. Those who reported diagnosed hypertension in China had an average VAS score of 73.04, which is comparable to 74.0 in South Asia [14], 77.2 in Indonesia [23], 63.7 in Nepal [22], and 63.9 in Pakistan [21].

The HRQoL of hypertensive people is associated with many factors. The effects of age, comorbidity, socioeconomic status (including education, income, employment, and marital status), region, and lifestyle (drinking and exercise) reached the threshold of MCID in this study. The HRQoL of people with self-reported diagnosed hypertension decreased with age. Those with other chronic conditions had even lower HRQoL. These results are consistent with findings of previous studies [12,25,27]. Although obesity can result in many chronic conditions, we found that underweight has a more profound negative effect on the HRQoL of hypertensive people than obesity, similar to the reports published elsewhere [44,58]. About 9% of respondents who reported diagnosed hypertension in this study were underweighted, compared with 1.66% with obesity. In general populations in China, men usually have higher HRQoL than women [24]. However, this study showed that women with self-reported diagnosed hypertension reported higher HRQoL than their male counterparts, despite a lack of clinical significance in terms of the MCID.

Socioeconomic disparities in the HRQoL of people with self-reported diagnosed hypertension are evident. Higher income, better education, and employment are associated with higher HRQoL. Marriage is also associated with higher HRQoL. There is a common belief that these factors shape HRQoL through access to material support, social participation, and opportunity to self-control over life [10,21,22,26,27,28]. Workforce and social participation are essential by itself for high HRQoL. Education is a key determinant of workforce and social participation. Better education can also improve health literacy, empowering consumers to better engage in self-care and health care services [59,60]. Marriage may provide additional benefits for the management of chronic conditions, which often requires significant changes in lifestyles. A study showed that older men benefit more from marriage in HRQoL [61].

There exist significant regional differences in the HRQoL of people who reported diagnosed hypertension. Those residing in the western (less developed) region have lower HRQoL compared with their better-off eastern counterparts. Such a difference persists after controlling for variations in other factors and remains clinically significant in terms of the MCID. Interestingly, rural people with self-reported diagnosed hypertension rated higher in VAS than their urban counterparts, despite having a statistically lower but clinically insignificant utility index. This illustrates the importance of localization of the population-preference value sets. Socioeconomic and cultural differences between urban and rural areas in China are still profound. Previous studies revealed inconsistent urban-rural differences in the EQ-5D-3L utility index and VAS scores in China using a value set derived from four big urban settings [10,41].

Smoking, alcohol consumption, and sedentary lifestyles are risk factors of hypertension and many other chronic conditions [62,63,64]. However, we found in this study that people who reported diagnosed hypertension and perceived lower HRQoL were less likely to smoke and drink. These results are consistent with findings of previous studies [26,54]. It may be attributable to the success of the management programs for chronic conditions [65,66,67]. Indeed, participation in the management programs for chronic conditions is a significant independent predictor of higher HRQoL as revealed in this study. This study also proved that regular exercises are associated with higher HRQoL in people with self-reported diagnosed hypertension. It important to note that the cross-sectional design of this study does not assume causal relationships.

In a country without universal health coverage, such as in China, low income can still impose a great barrier for patients to access health care services [68]. The Chinese government has placed high expectations on preventive measures for the development of a more cost-effective health care system. Indeed, as revealed in this study, the hypertensive people who enrolled in management programs for chronic conditions had higher utility index and VAS scores and experienced less pain/discomfort problems. Those who received health examinations reported less problems in mobility, self-care, and usual activities. Health examinations can help detect hypertension at an early stage, often without obvious symptoms. These preventive measures also help increase the awareness of patients on the importance of appropriate control of blood pressure [10]. However, a strong primary care system is essential to maximize the benefits of these medical interventions. The effect of the preventive care measures tested in this study failed to reach the threshold of MCID. This could be an indication of a shortage of effective follow-up services [11,13,49,69,70,71].

There are several limitations in this study. This study employed a cross-sectional design and no causal relationships can be assumed. The EQ-5D-3L used in this study is a validated instrument for measuring HRQoL in China, but it has high ceiling effects. It does not capture details in many aspects of HRQoL either. The NHSS collected self-reported data, which may lead to recall or reporting bias and data inaccuracies. Hypertensive conditions captured in the study were restricted to those self-reported cases diagnosed by doctors, which are subject to the influence of self-awareness [8,11,13]. Although such an approach has been widely adopted in health services studies [10,17,18,26,27], it is likely to lead to under-reporting. The calculation of BMI was also based on self-reported weight and height data, which can lead to certain level of inaccuracy. Due to limitations in data availability and completeness, a nominal measurement was applied for measuring comorbidities. The categorization of smoking and alcohol drinking was also simply and crude. These are a result of comprise of data granularity for large sample size. But the simple and crude categorization can help avoid exacerbating further bias of measurements resulting from a combination of incomplete data. Future studies should explore the impacts of BMI, smoking, drinking, and exercises on the HRQoL of hypertensive people using more objective and accurate measurements.

## 5. Conclusions

In conclusion, hypertension is associated with a lower HRQoL. This study provides a national representative estimate on the HRQoL of people with self-reported diagnosed hypertension in China, which can be used for calculating the burden of hypertension. Higher socioeconomic status and participation in management programs for chronic conditions are independent predictors of higher HRQoL of hypertensive people. These findings have some policy implications. A systems and integrated approach to the management of hypertension is critical. Priorities should be given to those who are old, poor, unemployed, and live in western and rural regions. Early interventions on unhealthy lifestyles remain a great challenge in China and warrant further studies.

## Figures and Tables

**Table 1:** Utility index and VAS scores of respondents with different characteristics.

Variable	Description	Respondents without Diagnosed Hypertension						Respondents with Diagnosed Hypertension						Percentage of Diagnosed Hypertension
		*n*	%	VAS		Utility		*n*	%	VAS		Utility		
				Mean	SD	Mean	SD			Mean	SD	Mean	SD	
**Biology/genetics**														
Gender														
	Male	75,798	47.77	82.94	12.74	0.989	0.047	14,032	46.68	74.09	15.15	0.966	0.088	15.62
	Female	82,859	52.23	81.91	13.11	0.988	0.047	16,031	53.32	72.12	15.01	0.962	0.088	16.21
Age (years)														
	15–24	14,079	8.87	90.41	8.34	0.998	0.022	15	0.05	82.67	15.80	0.969	0.101	0.11
	25–34	24,170	15.23	88.06	9.31	0.997	0.024	177	0.59	80.89	15.32	0.994	0.024	0.73
	35–44	33,571	21.16	84.94	11.08	0.995	0.029	1510	5.02	79.05	14.86	0.984	0.058	4.30
	45–54	35,693	22.50	82.06	12.32	0.992	0.037	5307	17.65	76.54	14.91	0.980	0.064	12.94
	55–64	30,457	19.20	78.33	13.11	0.985	0.050	10,075	33.51	74.15	14.40	0.974	0.071	24.86
	65–74	14,013	8.83	74.19	14.07	0.974	0.068	8125	27.03	71.17	14.82	0.959	0.090	36.70
	75+	6674	4.21	69.93	15.32	0.940	0.113	4854	16.15	67.84	15.23	0.923	0.127	42.11
BMI														
	Underweight (<18.5)	14,380	9.08	79.00	15.61	0.977	0.077	1574	5.24	66.53	16.49	0.928	0.131	9.87
	Normal range (18.5–24.9)	116,107	73.28	82.73	12.55	0.990	0.043	17,843	59.40	73.00	14.85	0.963	0.091	13.32
	Overweight (25.0–29.9)	25,325	15.98	82.92	12.59	0.990	0.041	9189	30.59	74.08	14.99	0.971	0.072	26.62
	Obese (≥30.0)	2629	1.66	81.58	14.03	0.986	0.055	1431	4.76	73.95	15.62	0.968	0.072	35.25
Comorbidity														
	No	138,022	86.99	84.05	11.63	0.993	0.036	19,592	65.17	75.84	13.87	0.976	0.069	12.43
	Yes	20,635	13.01	71.39	15.61	0.962	0.085	10,471	34.83	67.80	15.91	0.941	0.112	33.66
**Socioeconomic characteristics**														
Level of education														
	Illiterate	16,958	10.69	74.60	14.98	0.969	0.079	5751	19.13	68.79	15.83	0.939	0.115	25.33
	Primary school	39,579	24.95	79.58	13.54	0.985	0.054	9374	31.18	72.51	15.04	0.961	0.089	19.15
	Junior middle school	57,350	36.15	84.26	11.81	0.993	0.037	8527	28.36	74.93	14.50	0.973	0.073	12.94
	Senior middle school	27,835	17.54	84.85	11.60	0.994	0.033	4600	15.30	74.93	14.42	0.977	0.072	14.18
	University/college	16,935	10.67	86.50	10.24	0.997	0.021	1811	6.02	75.56	14.64	0.980	0.061	9.66
Income ranking														
	<percentile 20	29,331	18.50	79.43	14.86	0.981	0.062	6371	21.20	69.65	16.16	0.948	0.103	17.85
	percentile 20–39.9	30,082	18.97	81.97	13.11	0.988	0.049	5389	17.94	72.45	15.11	0.962	0.089	15.19
	percentile 40–59.9	31,581	19.91	83.03	12.48	0.990	0.044	5543	18.45	73.68	14.66	0.967	0.083	14.93
	percentile 60–79.9	32,967	20.79	83.33	12.12	0.992	0.039	6117	20.36	74.33	14.51	0.969	0.082	15.65
	≥percentile 80	34,626	21.83	83.84	11.75	0.992	0.038	6625	22.05	75.04	14.38	0.972	0.077	16.06
Employment														
	Employed	11,4748	72.32	83.80	11.84	0.993	0.031	12,866	42.80	75.86	14.32	0.978	0.061	10.08
	Retired	16,942	10.68	77.50	13.17	0.982	0.062	10,332	34.37	72.66	14.24	0.965	0.085	37.88
	Student	4730	2.98	91.12	7.95	0.999	0.016	17	0.06	70.59	15.19	0.977	0.050	0.36
	Unemployed	22,237	14.02	77.07	15.92	0.970	0.085	6848	22.78	68.33	16.54	0.934	0.122	23.55
Marital status														
	Never married/Single	16,731	10.55	88.54	10.73	0.994	0.040	400	1.33	69.57	16.70	0.952	0.113	2.34
	Married	13,1058	82.61	82.24	12.64	0.990	0.043	24,697	82.15	73.89	14.95	0.969	0.082	15.86
	Widowed	8362	5.27	73.11	14.89	0.961	0.089	4570	15.20	68.85	15.00	0.938	0.110	35.34
	Divorced	2502	1.58	80.83	14.12	0.986	0.056	396	1.32	71.87	16.00	0.963	0.081	13.67
**Communities and regions**														
Residency														
	Urban	77,129	48.61	82.31	12.90	0.990	0.045	16,935	56.33	72.79	14.90	0.966	0.084	18.00
	Rural	81,528	51.39	82.49	12.98	0.988	0.049	13,128	43.67	73.36	15.37	0.961	0.092	13.87
Region														
	Eastern	54,155	34.13	83.78	12.29	0.991	0.044	12,420	41.31	74.88	14.74	0.968	0.083	18.66
	Central	48,661	30.67	82.19	13.19	0.988	0.048	9645	32.08	72.50	15.04	0.963	0.090	16.54
	Western	55,841	35.20	81.25	13.21	0.988	0.048	7998	26.60	70.83	15.41	0.957	0.092	12.53
**Health behaviors**														
Smoking														
	No	116,204	73.29	82.30	13.10	0.988	0.049	23,195	77.19	72.45	15.27	0.960	0.093	16.64
	Yes	42,355	26.71	82.66	12.50	0.990	0.040	6853	22.81	75.04	14.35	0.975	0.067	13.93
Drinking														
	No	120,987	76.26	82.16	13.21	0.988	0.050	23,719	78.90	72.01	15.31	0.959	0.095	16.39
	Yes	37,662	23.74	83.18	12.01	0.992	0.033	6343	21.10	76.91	13.67	0.982	0.052	14.41
Regular exercise														
	No	114,623	72.45	82.27	13.19	0.988	0.051	17,749	59.16	72.04	15.80	0.954	0.104	13.41
	Yes	43,591	27.55	82.76	12.27	0.992	0.032	12,252	40.84	74.48	13.91	0.978	0.053	21.94
Total		158,657	100.00	82.40	12.94	0.989	0.047	30,063	100.00	73.04	15.11	0.964	0.088	15.93

**Table 2:** Percentage of reported problems on the five dimensions of EQ-5D-3L.

Dimension		Respondents without Diagnosed Hypertension		Respondents with Diagnosed Hypertension		χ²	*p*
		*n*	%	*n*	%		
Mobility	No problems	152,134	95.89	25,533	84.93	5500.00	<0.001
	Some problems	6166	3.89	4268	14.20		
	Confined to bed	357	0.23	262	0.87		
Self-care	No problems	155,298	97.88	27,657	92.00	3000.00	<0.001
	Some problems	2930	1.85	2028	6.75		
	Unable to	429	0.27	378	1.26		
Usual activities	No problems	153,446	96.72	26,520	88.21	4200.00	<0.001
	Some problems	4371	2.75	2853	9.49		
	Unable to	840	0.53	690	2.30		
Pain/discomfort	No problems	142,661	89.92	22,259	74.04	5800.00	<0.001
	Some problems	15,409	9.71	7419	24.68		
	Extreme problems	587	0.37	385	1.28		
Anxiety/depression	No problems	151,795	95.67	26,972	89.72	1800.00	<0.001
	Some problems	6536	4.12	2926	9.73		
	Extreme problems	326	0.21	165	0.55		

**Table 3:** Association between self-reported diagnosed hypertension and reported problems on the five dimensions: logistic regression analyses adjusting for variations of multiple factors (*n* = 188,720).

Variables		Mobility			Self-Care			Usual Activities			Pain/Discomfort			Anxiety/Depression		
		AOR	95% CI		AOR	95% CI		AOR	95% CI		AOR	95% CI		AOR	95% CI	
**Hypertension**																
	No (reference)															
	Yes	1.70	1.62	1.78	1.70	1.59	1.81	1.67	1.58	1.76	1.43	1.38	1.48	1.47	1.39	1.55
**Biology/Genetics**																
Gender																
	Male (reference)															
	Female	0.71	0.67	0.75	0.60	0.56	0.64	0.63	0.60	0.67	1.17	1.13	1.22	1.04	0.98	1.10
Age (years)																
	15–24 (reference)															
	25–34	1.80	1.29	2.51	1.82	1.24	2.67	1.85	1.34	2.55	1.66	1.36	2.01	2.22	1.76	2.80
	35–44	3.63	2.65	4.98	3.35	2.32	4.82	3.50	2.57	4.76	3.79	3.15	4.56	4.50	3.59	5.63
	45–54	6.48	4.76	8.83	5.11	3.58	7.30	5.71	4.22	7.73	6.27	5.22	7.54	5.49	4.38	6.87
	55–64	9.39	6.89	12.80	6.56	4.59	9.38	7.25	5.35	9.81	8.75	7.27	10.53	6.34	5.06	7.95
	65–74	15.01	10.99	20.50	9.93	6.93	14.23	11.27	8.30	15.31	11.03	9.14	13.30	6.35	5.04	7.99
	75+	32.56	23.78	44.57	19.98	13.90	28.70	23.52	17.27	32.03	15.06	12.43	18.24	7.96	6.29	10.09
BMI																
	Underweight (<18.5) (reference)															
	Normal range (18.5–24.9)	0.72	0.67	0.77	0.71	0.66	0.78	0.71	0.66	0.76	0.76	0.73	0.80	0.72	0.67	0.77
	Overweight (25.0–29.9)	0.82	0.76	0.89	0.71	0.64	0.79	0.69	0.63	0.76	0.82	0.77	0.87	0.65	0.60	0.70
	Obese (≥30.0)	1.15	1.00	1.32	0.87	0.72	1.05	0.89	0.76	1.04	0.95	0.85	1.06	0.72	0.62	0.84
Comorbidity																
	No (reference)															
	Yes	3.10	3.24	2.96	2.77	2.94	2.61	3.31	3.48	3.14	3.94	4.07	3.81	3.36	3.52	3.21
**Socioeconomic characteristics**																
Level of education																
	Illiterate (reference)															
	Primary school	0.83	0.78	0.88	0.76	0.70	0.82	0.73	0.68	0.77	0.86	0.83	0.90	0.78	0.74	0.83
	Junior middle school	0.71	0.66	0.76	0.65	0.59	0.71	0.62	0.58	0.67	0.70	0.67	0.74	0.71	0.66	0.76
	Senior middle school	0.59	0.54	0.64	0.54	0.47	0.61	0.52	0.47	0.58	0.66	0.62	0.70	0.68	0.62	0.74
	University/college	0.49	0.42	0.56	0.47	0.39	0.56	0.43	0.37	0.50	0.60	0.55	0.66	0.74	0.65	0.83
Income ranking																
	<percentile 20 (reference)															
	percentile 20–39.9	0.82	0.77	0.87	0.83	0.76	0.90	0.80	0.75	0.86	0.81	0.78	0.85	0.80	0.75	0.85
	percentile 40–59.9	0.72	0.67	0.77	0.75	0.69	0.82	0.71	0.66	0.76	0.77	0.74	0.81	0.70	0.66	0.75
	percentile 60–79.9	0.67	0.63	0.72	0.70	0.64	0.76	0.66	0.61	0.71	0.70	0.66	0.73	0.64	0.60	0.69
	≥percentile 80	0.65	0.61	0.70	0.69	0.63	0.75	0.65	0.60	0.70	0.67	0.64	0.70	0.60	0.56	0.65
Employment																
	Employed (reference)															
	Retired	1.92	1.78	2.07	2.60	2.35	2.88	2.21	2.03	2.40	1.12	1.06	1.18	1.03	0.95	1.11
	Student	0.74	0.42	1.30	0.81	0.42	1.57	0.54	0.29	1.02	0.57	0.40	0.81	0.63	0.43	0.94
	Unemployed	2.69	2.54	2.85	3.40	3.14	3.67	3.15	2.96	3.36	1.60	1.53	1.67	1.72	1.62	1.82
Marital status																
	Never married/Single (reference)															
	Married	0.52	0.46	0.60	0.46	0.39	0.54	0.45	0.40	0.52	0.86	0.78	0.95	0.59	0.53	0.66
	Widowed	0.64	0.56	0.74	0.58	0.49	0.69	0.55	0.48	0.64	1.00	0.90	1.12	0.73	0.64	0.83
	Divorced	0.85	0.69	1.05	0.79	0.60	1.03	0.77	0.62	0.97	1.23	1.05	1.43	1.16	0.97	1.39
**Communities and regions**																
Residency																
	Urban (reference)															
	Rural	1.08	1.02	1.13	1.18	1.10	1.26	1.16	1.10	1.23	1.00	0.96	1.03	1.03	0.98	1.08
Region																
	Eastern (reference)															
	Central	1.21	1.15	1.28	1.21	1.12	1.29	1.17	1.10	1.25	1.28	1.23	1.33	1.34	1.27	1.41
	Western	1.43	1.36	1.51	1.42	1.32	1.52	1.48	1.39	1.57	1.41	1.36	1.47	1.60	1.51	1.68
**Health behaviors**																
Smoking																
	No (reference)															
	Yes	0.82	0.87	0.77	0.73	0.79	0.67	0.76	0.81	0.71	0.96	1.00	0.92	0.92	0.98	0.86
Drinking																
	No (reference)															
	Yes	0.74	0.79	0.69	0.53	0.58	0.48	0.60	0.65	0.56	1.00	1.05	0.96	0.91	0.97	0.86
Regular exercise																
	No (reference)															
	Yes	0.49	0.52	0.46	0.41	0.44	0.37	0.45	0.48	0.42	0.80	0.83	0.77	0.73	0.78	0.69

**Table 4:** Association of self-reported diagnosed hypertension with health utility and VAS scores: regression analyses adjusting for variations of multiple factors (*n* = 188,720).

Variables		Tobit Regression on Utility Index					Linear Regression on VAS				
		β	SE	*p*	95% CI		β	SE	*p*	95% CI	
**Hypertension**											
	No (reference)										
	Yes	−0.04	0.00	<0.001	−0.04	−0.04	−3.22	0.10	<0.001	−3.41	−3.03
**Biology/Genetics**											
Gender											
	Male (reference)										
	Female	0.00	0.00	0.009	0.00	0.01	−0.09	0.07	0.205	−0.23	0.05
Age (years)											
	15–24 (reference)										
	25–34	−0.05	0.01	<0.001	−0.06	−0.04	−2.29	0.12	<0.001	−2.53	−2.06
	35–44	−0.10	0.01	<0.001	−0.11	−0.09	−4.99	0.13	<0.001	−5.25	−4.74
	45–54	−0.13	0.01	<0.001	−0.15	−0.12	−7.50	0.13	<0.001	−7.75	−7.24
	55–64	−0.16	0.01	<0.001	−0.17	−0.15	−9.57	0.14	<0.001	−9.84	−9.29
	65–74	−0.18	0.01	<0.001	−0.19	−0.17	−11.80	0.16	<0.001	−12.12	−11.48
	75+	−0.24	0.01	<0.001	−0.25	−0.23	−14.06	0.21	<0.001	−14.46	−13.65
BMI											
	Underweight (<18.5) (reference)										
	Normal range (18.5–24.9)	0.03	0.00	<0.001	0.03	0.03	2.27	0.11	<0.001	2.06	2.48
	Overweight (25.0–29.9)	0.03	0.00	<0.001	0.02	0.03	2.50	0.13	<0.001	2.26	2.75
	Obese (≥30.0)	0.01	0.00	0.044	0.00	0.02	1.32	0.23	<0.001	0.86	1.78
Comorbidity											
	No (reference)										
	Yes	−0.12	0.00	<0.001	−0.12	−0.11	−8.39	0.09	<0.001	−8.57	−8.20
**Socioeconomic characteristics**											
Level of education											
	Illiterate (reference)										
	Primary school	0.02	0.00	<0.001	0.01	0.02	1.88	0.11	<0.001	1.66	2.10
	Junior middle school	0.04	0.00	<0.001	0.03	0.04	3.07	0.12	<0.001	2.84	3.30
	Senior middle school	0.04	0.00	<0.001	0.04	0.05	2.93	0.13	<0.001	2.67	3.18
	University/college	0.04	0.00	<0.001	0.04	0.05	2.71	0.14	<0.001	2.43	2.99
Income ranking											
	<percentile 20 (reference)										
	percentile 20–39.9	0.02	0.00	<0.001	0.02	0.02	1.30	0.09	<0.001	1.11	1.48
	percentile 40–59.9	0.03	0.00	<0.001	0.02	0.03	1.94	0.09	<0.001	1.76	2.12
	percentile 60–79.9	0.03	0.00	<0.001	0.03	0.04	2.25	0.09	<0.001	2.07	2.43
	≥percentile 80	0.04	0.00	<0.001	0.03	0.04	2.69	0.09	<0.001	2.51	2.87
Employment											
	Employed (reference)										
	Retired	−0.03	0.00	<0.001	−0.03	−0.02	−1.07	0.11	<0.001	−1.29	−0.85
	Student	0.03	0.01	0.004	0.01	0.05	0.87	0.15	<0.001	0.57	1.17
	Unemployed	−0.06	0.00	<0.001	−0.07	−0.06	−3.01	0.10	<0.001	−3.20	−2.82
Marital status											
	Never married/Single (reference)										
	Married	0.03	0.00	<0.001	0.03	0.04	0.20	0.12	0.089	−0.03	0.44
	Widowed	0.02	0.00	<0.001	0.01	0.02	−0.74	0.18	<0.001	−1.10	−0.38
	Divorced	−0.01	0.01	0.250	−0.02	0.00	−1.76	0.27	<0.001	−2.29	−1.24
**Communities and regions**											
Residency											
	Urban (reference)										
	Rural	0.00	0.00	0.139	0.00	0.00	0.72	0.06	<0.001	0.60	0.84
Region											
	Eastern (reference)										
	Central	−0.02	0.00	<0.001	−0.02	−0.01	−1.35	0.07	<0.001	−1.48	−1.22
	Western	−0.03	0.00	<0.001	−0.03	−0.03	−2.58	0.07	<0.001	−2.71	−2.46
**Health behaviors**											
Smoking											
	No (reference)										
	Yes	0.01	0.00	<0.001	0.01	0.01	0.19	0.08	0.012	0.04	0.34
Drinking											
	No (reference)										
	Yes	0.01	0.00	<0.001	0.00	0.01	0.75	0.07	<0.001	0.61	0.90
Regular exercise											
	No (reference)										
	Yes	0.03	0.00	<0.001	0.03	0.04	1.09	0.07	<0.001	0.95	1.22

**Table 5:** Factors associated with reported problems on the five dimensions in the respondents with self-reported diagnosed hypertension: results of logistic regression analyses (*n* = 30,063).

Variables		Mobility			Self-Care			Usual Activities			Pain/Discomfort			Anxiety/Depression		
		AOR	95%CI		AOR	95%CI		AOR	95%CI		AOR	95%CI		AOR	95%CI	
**Biology/Genetics**																
Gender																
	Male (reference)															
	Female	0.66	0.61	0.72	0.57	0.51	0.63	0.60	0.55	0.66	1.08	1.00	1.16	0.99	0.90	1.10
Age (years)																
	15–24 (reference)															
	25–34	0.19	0.01	3.79	0.73	0.05	10.39	0.50	0.06	4.36	1.57	0.17	14.29	0.54	0.12	2.38
	35–44	1.62	0.17	15.65	1.28	0.12	13.81	1.73	0.33	9.03	3.24	0.38	27.51	1.01	0.27	3.78
	45–54	2.20	0.23	21.00	1.38	0.13	14.72	2.21	0.43	11.30	3.63	0.43	30.64	0.89	0.24	3.29
	55–64	2.78	0.29	26.49	1.44	0.14	15.30	2.47	0.48	12.59	4.39	0.52	37.08	0.84	0.23	3.10
	65–74	4.50	0.47	42.90	2.21	0.21	23.45	3.92	0.77	20.01	5.37	0.64	45.42	0.88	0.24	3.25
	75+	9.36	0.98	89.30	4.06	0.38	43.13	7.92	1.55	40.40	7.59	0.90	64.22	1.06	0.28	3.93
BMI																
	Underweight (<18.5) (reference)															
	Normal range (18.5–24.9)	0.82	0.72	0.94	0.81	0.69	0.96	0.79	0.68	0.91	0.84	0.75	0.95	0.79	0.68	0.91
	Overweight (25.0–29.9)	0.92	0.79	1.06	0.75	0.63	0.90	0.74	0.63	0.86	0.94	0.83	1.06	0.72	0.61	0.85
	Obese (≥30.0)	1.19	0.97	1.47	0.86	0.66	1.13	0.87	0.69	1.10	1.01	0.85	1.20	0.67	0.52	0.85
Comorbidity																
	No (reference)															
	Yes	2.56	2.75	2.39	2.39	2.62	2.18	2.68	2.90	2.48	2.68	2.83	2.53	2.57	2.79	2.38
**Socioeconomic characteristics**																
Level of education																
	Illiterate (reference)															
	Primary school	0.89	0.81	0.97	0.77	0.68	0.87	0.72	0.65	0.80	0.86	0.79	0.93	0.82	0.74	0.91
	Junior middle school	0.77	0.68	0.86	0.64	0.55	0.74	0.65	0.57	0.74	0.73	0.67	0.80	0.76	0.67	0.86
	Senior middle school	0.63	0.55	0.73	0.58	0.48	0.70	0.58	0.49	0.67	0.70	0.62	0.78	0.72	0.61	0.84
	University/college	0.55	0.45	0.68	0.55	0.41	0.72	0.55	0.43	0.69	0.66	0.56	0.77	0.73	0.58	0.93
Income ranking																
	<percentile 20 (reference)															
	percentile 20–39.9	0.84	0.75	0.93	0.89	0.78	1.01	0.89	0.79	1.00	0.82	0.75	0.89	0.82	0.73	0.91
	percentile 40–59.9	0.76	0.68	0.84	0.80	0.69	0.92	0.79	0.70	0.90	0.81	0.74	0.88	0.66	0.58	0.74
	percentile 60–79.9	0.73	0.66	0.81	0.76	0.66	0.88	0.72	0.64	0.82	0.74	0.67	0.80	0.67	0.60	0.76
	≥percentile 80	0.69	0.62	0.77	0.72	0.62	0.83	0.75	0.66	0.85	0.69	0.63	0.75	0.60	0.53	0.68
Employment																
	Employed (reference)															
	Retired	1.83	1.62	2.06	2.54	2.16	2.97	2.10	1.84	2.40	1.18	1.08	1.29	1.16	1.02	1.33
	Student	1.25	0.29	5.39	1.73	0.19	15.55	2.10	0.36	12.25	0.80	0.23	2.73	0.82	0.09	7.24
	Unemployed	2.55	2.31	2.81	3.25	2.85	3.69	2.93	2.63	3.27	1.62	1.50	1.74	1.73	1.56	1.92
Marital status																
	Never married/Single (reference)															
	Married	0.66	0.50	0.88	0.70	0.49	1.00	0.63	0.47	0.85	0.87	0.69	1.10	0.70	0.52	0.95
	Widowed	0.79	0.58	1.06	0.82	0.57	1.19	0.76	0.56	1.04	0.97	0.76	1.25	0.84	0.62	1.15
	Divorced	1.11	0.73	1.69	1.18	0.70	2.00	1.16	0.75	1.81	1.25	0.90	1.75	1.40	0.93	2.11
**Communities and regions**																
Residency																
	Urban (reference)															
	Rural	1.10	1.01	1.19	1.21	1.08	1.34	1.20	1.09	1.32	1.09	1.02	1.17	1.22	1.11	1.34
Region																
	Eastern (reference)															
	Central	1.20	1.10	1.30	1.18	1.05	1.31	1.16	1.06	1.28	1.34	1.26	1.43	1.45	1.32	1.60
	Western	1.49	1.37	1.62	1.45	1.30	1.62	1.51	1.38	1.66	1.60	1.49	1.71	1.92	1.75	2.12
**Health behaviors**																
Smoking																
	No (reference)															
	Yes	0.78	0.87	0.71	0.69	0.79	0.60	0.69	0.78	0.61	0.89	0.97	0.82	0.88	0.98	0.78
Drinking																
	No (reference)															
	Yes	0.62	0.69	0.55	0.43	0.51	0.37	0.52	0.59	0.45	0.82	0.90	0.76	0.74	0.84	0.65
Regular exercise																
	No (reference)															
	Yes	0.45	0.49	0.41	0.36	0.41	0.32	0.40	0.45	0.36	0.71	0.76	0.66	0.62	0.69	0.57
**Preventive care services**																
Management program																
	No (reference)															
	Yes	0.89	0.83	0.97	0.98	0.89	1.09	0.90	0.83	0.98	0.87	0.82	0.93	0.97	0.88	1.05
Health examination																
	No (reference)															
	Yes	0.81	0.76	0.87	0.70	0.64	0.77	0.74	0.69	0.80	0.96	0.91	1.02	0.91	0.84	0.98

**Table 6:** Factors associated with utility index and VAS scores of respondents with self-reported diagnosed hypertension: results of multivariate regression analyses (*n* = 30,063).

Variables		Tobit Regression on Utility Index					Linear Regression on VAS				
		β	SE	*p*	95%CI		β	SE	*p*	95%CI	
**Biology/Genetics**											
Gender											
	Male (reference)										
	Female	0.02	0.00	<0.001	0.02	0.03	0.59	0.21	0.006	0.17	1.01
Age (years)											
	15–24 (reference)										
	25–34	**−0.01**	0.09	0.936	−0.18	0.16	−7.72	3.36	0.021	−14.30	−1.14
	35–44	**−0.07**	0.09	0.426	−0.23	0.10	−9.37	3.20	0.003	−15.63	−3.10
	45–54	**−0.08**	0.08	0.359	−0.24	0.09	−11.29	3.18	<0.001	−17.53	−5.05
	55–64	**−0.09**	0.08	0.289	−0.26	0.08	−12.38	3.18	<0.001	−18.62	−6.15
	65–74	**−0.12**	0.08	0.173	−0.28	0.05	−13.98	3.18	<0.001	−20.22	−7.74
	75+	**−0.17**	0.09	0.041	−0.34	−0.01	−15.89	3.19	<0.001	−22.14	−9.64
BMI											
	Underweight (<18.5) (reference)										
	Normal range (18.5–24.9)	0.02	0.01	<0.001	0.01	0.04	3.03	0.41	<0.001	2.23	3.84
	Overweight (25.0–29.9)	0.02	0.01	<0.001	0.01	0.03	2.96	0.43	<0.001	2.12	3.81
	Obese (≥30.0)	0.01	0.01	0.274	−0.01	0.02	2.77	0.56	<0.001	1.66	3.88
Comorbidity											
	No (reference)										
	Yes	**−0.10**	0.00	<0.001	−0.10	−0.09	−6.99	0.18	<0.001	−7.34	−6.64
**Socioeconomic characteristics**											
Level of education											
	Illiterate (reference)										
	Primary school	**0.02**	0.00	<0.001	0.01	0.03	1.62	0.26	<0.001	1.12	2.12
	Junior middle school	**0.03**	0.00	<0.001	0.02	0.04	2.16	0.28	<0.001	1.61	2.72
	Senior middle school	**0.04**	0.01	<0.001	0.03	0.05	1.80	0.33	<0.001	1.15	2.45
	University/college	**0.05**	0.01	<0.001	0.03	0.06	1.96	0.44	<0.001	1.11	2.82
Income ranking											
	<percentile 20 (reference)										
	percentile 20–39.9	**0.02**	0.00	<0.001	0.01	0.03	1.63	0.27	<0.001	1.10	2.16
	percentile 40–59.9	**0.03**	0.00	<0.001	0.02	0.03	2.42	0.27	<0.001	1.89	2.94
	percentile 60–79.9	**0.03**	0.00	<0.001	0.02	0.04	2.90	0.27	<0.001	2.38	3.42
	≥percentile 80	**0.04**	0.00	<0.001	0.03	0.04	3.31	0.27	<0.001	2.79	3.84
Employment											
	Employed (reference)										
	Retired	**−0.04**	0.00	<0.001	−0.05	−0.03	−2.11	0.26	<0.001	−2.61	−1.61
	Student	**−0.01**	0.05	0.759	−0.11	0.08	−5.99	2.73	0.028	−11.34	−0.65
	Unemployed	**−0.08**	0.00	<0.001	−0.08	−0.07	−4.41	0.25	<0.001	−4.90	−3.92
Marital status											
	Never married/Single (reference)										
	Married	**0.03**	0.01	0.006	0.01	0.05	3.71	0.77	<0.001	2.20	5.22
	Widowed	**0.02**	0.01	0.162	−0.01	0.04	2.98	0.80	<0.001	1.41	4.55
	Divorced	**−0.01**	0.02	0.579	−0.04	0.02	1.20	1.05	0.253	−0.86	3.26
**Communities and regions**											
Residency											
	Urban (reference)										
	Rural	−0.01	0.00	0.021	−0.01	0.00	0.67	0.20	0.001	0.28	1.07
Region											
	Eastern (reference)										
	Central	**−0.02**	0.00	<0.001	−0.03	−0.02	−2.33	0.19	<0.001	−2.70	−1.96
	Western	**−0.04**	0.00	<0.001	−0.05	−0.03	−3.78	0.20	<0.001	−4.18	−3.38
**Health behaviors**											
Smoking											
	No (reference)										
	Yes	0.02	0.00	<0.001	0.02	0.03	0.46	0.22	0.039	0.02	0.90
Drinking											
	No (reference)										
	Yes	**0.04**	0.00	<0.001	0.03	0.04	2.21	0.22	<0.001	1.78	2.65
Regular exercise											
	No (reference)										
	Yes	**0.06**	0.00	<0.001	0.05	0.07	2.73	0.19	<0.001	2.35	3.11
**Preventive care services**											
Management program											
	No (reference)										
	Yes	0.01	0.00	<0.001	0.01	0.02	1.02	0.19	<0.001	0.66	1.39
Health examination											
	No (reference)										
	Yes	0.02	0.00	<0.001	0.01	0.02	0.38	0.17	0.026	0.05	0.71

Bold figures indicate the effects of the factors reached the threshold of MCID.
